# Retinal Nerve Fiber Layer Thicknesses in Three Different Optic Nerve Head Size Groups Measured by Cirrus Spectral Domain Optical Coherence Tomography

**DOI:** 10.4274/tjo.29567

**Published:** 2016-04-05

**Authors:** Sirel Gür Güngör, Ahmet Akman, Ali Küçüködük, Meriç Çolak

**Affiliations:** 1 Başkent University Faculty of Medicine, Department of Ophthalmology, Ankara, Turkey; 2 Başkent University Faculty of Health Sciences, Ankara, Turkey

**Keywords:** Retinal nerve fiber layer, optic nerve head, optical coherence tomography

## Abstract

**Objectives:**

To compare the retinal nerve fiber layer (RNFL) thicknesses in three different optic nerve head (ONH) size groups measured by Cirrus spectral domain optical coherence tomography (OCT).

**Materials and Methods:**

Between January and March 2013, 253 eyes of 253 healthy subjects were enrolled in this study (mean age: 42.7±7.4 years [28-62 years]; 121 men and 132 women). The patients were divided into 3 groups according to ONH size: 77 patients in the “small ONH” group (ONH area <1.63 mm^2^), 90 patients in the “medium ONH” group (ONH area 1.63-1.97 mm^2^), and 86 patients in the “large ONH” group (ONH area >1.97 mm^2^).

**Results:**

There were significant differences in superior (p=0.008), inferior (p=0.004) and average RNFL thickness (p=0.001) between the small, medium and large ONH groups. Positive correlations between ONH size and inferior/average RNFL thicknesses were significant but very weak (r=0.150, p=0.017 and r=0.157, p=0.013 respectively).

**Conclusion:**

RNFL thickness as measured by Cirrus OCT is positively correlated with ONH size and the differences in RNFL thickness were statistically significant between groups. This correlation and difference may be the result of a varying distance between the circular scan and the ONH margin.

## INTRODUCTION

Although the assessment of peripapillary retinal nerve fiber layer (RNFL) thickness is essential in the diagnosis and management of glaucoma, its objective evaluation remains a challenge in clinical practice.^1^ Quantitative measurements of RNFL thickness have become possible with the development of imaging technologies such as optical coherence tomography (OCT).

Several authors have shown that the Cirrus HD Spectral Domain OCT has very good intra-observer repeatability in both healthy and glaucomatous eyes.^2,3,4^ The principle involved in image acquisition is similar for all the devices and involves a scan with a diode laser that collects information of RNFL thickness in a 3.4-mm-diameter circle centered on the optic nerve head (ONH).

Several investigators have reported that a larger ONH had more optic nerve fibers as determined histologically in human eyes.^5,6^ However, another histological study on human eyes could not detect a correlation between axon count and scleral canal area.^7^ studies using Stratus OCT (time domain OCT) have shown that eyes with large disc area have a thicker RNFL,^8,9^ whereas others did not find such a correlation.10 This study was undertaken to compare the RNFL thicknesses in three different ONH size groups as measured by Cirrus spectral domain OCT.

## MATERIALS AND METHODS

Between January and March 2013, 253 eyes of 253 subjects were enrolled in this study (mean age: 42.7±7.4 years [28-62 years], 121 men and 132 women). The study population consisted of consecutive patients with minor refractive disorders. All individuals underwent a complete ophthalmological examination, including visual acuity measurement, intraocular pressure measurement, slit-lamp biomicroscopy, and indirect ophthalmoscopy, to determine eligibility. Inclusion criteria were: best corrected visual acuity above 20/25, spherical refractive error between -5 and +5 diopters, cylindrical refractive error between -2 and +2 diopters, normal intraocular pressure ≤21 mmHg, normal appearance of the optic disc, no significant ocular disease found by routine ophthalmological examination, no history of glaucoma in the family, and no systemic diseases with possible ocular involvement, such as diabetes mellitus.

All participants gave their informed consent. The study was conducted according to the Declaration of Helsinki principles and was approved by the internal review board of the Başkent University Faculty of Medicine.

The patients were divided into 3 groups according to ONH area: 77 patients were in the ‘small ONH’ group (ONH area smaller than 1.63 mm^2^), 90 patients were in the ‘medium ONH’ group (ONH area between 1.63 mm^2^ and 1.97 mm^2^), and 86 patients were in the ‘large ONH’ group (ONH area larger than 1.97 mm^2^). These groups were classified according to the normal values and limits of the optic ONH parameters presented in Cirrus HD spectral domain OCT software.

### Optical Coherence Tomography Measurements

Cirrus HD spectral domain OCT (Carl Zeiss Meditec, Dublin, CA, USA) was used to measure both the peripapillary RNFL thickness and ONH area. The examination was performed under mydriasis by two experienced operators (S.G. and A.A.).

After pupil dilation, 6x6 mm cube optic disc scans, which were formed from 200 A scans for each of 200 B scans, were obtained. From this cube of data, the machine automatically identified the center of the disc and created a 3.4 mm calculation circle around the disc. The RNFL thickness along this peripapillary circle was analyzed and compared to normative data.

Ultimately, the signal strength had to be 6 or higher.

### Statistical Analysis

Statistical significances of RNFL thicknesses in ONH size groups (classified as small, medium and large) were analyzed with one-way ANOVA. Tukey’s post hoc test was used to identify which RNFL quadrants resulted in significant mean thickness differences between ONH size groups. Possible correlations between the RNFL thickness and ONH parameters were analyzed with Pearson correlation coefficient. All statistical analysis was done with Statistical Package for the Social Sciences version 15 (SPSS Inc., Chicago, IL, USA). The level of significance was taken as 0.05 in all statistical tests.

## RESULTS

The mean age was 40.12±8.49 years (range, 35-55 years) in the ‘small ONH’ group, 43.21±4.43 (range, 35-54 years) in the ‘medium ONH’ group and 41.07±12.42 years (range, 36-55 years) in the ‘large ONH’ group. There were no significant age differences between the groups (p=0.98). The male/female distribution was similar in all groups (p=0.69). There was no statistically significant difference in the mean refractive error between groups (p=0.87).

Significant differences in superior (p=0.008), inferior (p=0.004) and average RNFL thickness (p=0.001) were detected between the small, medium and large ONH groups. Mean values and standard deviations of all RNFL thickness parameters in each ONH size group are shown in Table 1. In general, larger ONH size corresponded to increased RNFL thicknesses in all quadrants.

**Figure 1:**
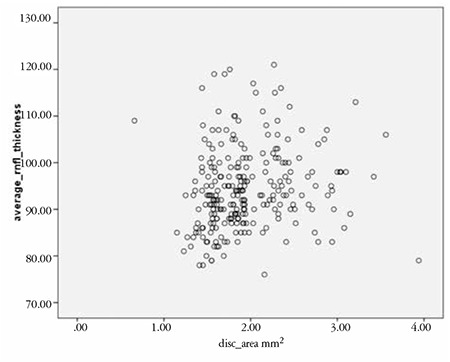
Scatter plot showing the correlation between average retinal nerve fiber layer thickness and optic nerve head area. RNFL: Retinal nerve fiber layer

**Figure 2:**
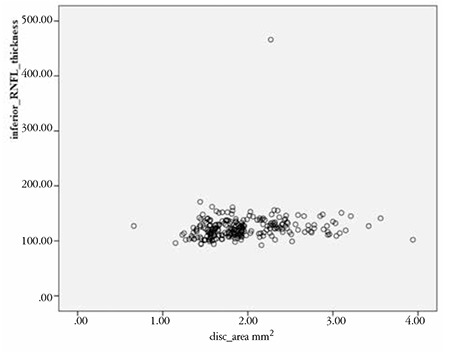
Scatter plot showing the correlation between inferior retinal nerve fiber layer thickness and optic nerve head area. RNFL: Retinal nerve fiber layer

The results of the correlation analysis of RNFL thickness and ONH size are reported in Table 2. Inferior and average RNFL thicknesses were positively correlated with ONH size; the correlations were significant correlations but very weak (r=0.150, p=0.017 and r=0.157, p=0.013, respectively). Figures 1 and 2 are scatter plots illustrating the correlations between average and inferior RNFL thickness and ONH area, respectively.

## DISCUSSION

To assess RNFL thickness, a circular scan concentric to the ONH is performed. In 1996 Schuman et al.^11^ found a circle diameter of 3.4 mm to be the most accurate in terms of reproducibility and all studies since then have used circular scans with this diameter, independently of ONH size. However, it is generally recognized that optic disc size shows a high inter-individual variability in normal eyes and its area may range between 0.8 and 6.0 mm^2^.^12^ Therefore, using a fixed-diameter circular scan in all eyes may result in RNFL thickness measurements performed at different distances from ONH margin.^8^

In our study, we obtained significant differences for superior, inferior and average RNFL thickness between ONH size groups. There were weak but significant positive correlations between ONH size and inferior and average RNFL thickness. In addition, we observed that the RNFL thicknesses in all quadrants increased with ONH size.

Savini et al.^8^ showed that RNFL thickness measured by Stratus OCT is positively correlated with ONH size, as determined by measurements of its area and diameter. The authors detected this correlation in the superior, inferior and nasal quadrants; in the temporal quadrant they detected a similar trend, but it did not reach statistical significance. They suggested such a correlation may be the result of either an increased number of nerve fibers in the eyes with larger discs or an artifact produced by the use of a fixed-diameter scan. The latter hypothesis was derived from the notion that if a fixed-diameter circular scan is used, the distance between the scan and the ONH margin will be reduced in the presence of a large ONH, which would lead to overestimation of RNFL thickness in patients with large ONH as the measurement would be made closer to the optic disc edge. In a subsequent study by Savini et al.,^9^ RNFL thickness was measured with a fixed 3.4 mm diameter circular scan and 2 customized-diameter scans (at 0.5 mm and 1 mm from the ONH edge) in 81 healthy subjects by Stratus OCT. It was found that when a fixed-diameter circular scan is used, larger discs show higher values; conversely, when the diameter is adjusted on the basis of ONH size, larger discs show lower values.

In a study by Kaushik et al.,^10^ the peripapillary RNFL of 32 normal eyes was scanned with the fast-scanning protocol at a diameter of 3.4 mm using Stratus OCT and disc area did not affect RNFL thickness measurement. They suggested that RNFL thickness is dependent on the distance from the center of the optic disc rather than the point of exit from the scleral canal and that RNFL thickness should be measured at similar distances from center of the optic disc, regardless of the size of scleral canal.

There are a few studies on this subject using spectral OCT. Mansoori et al.^13^ examined 65 healthy eyes using spectral OCT/SLO (scanning laser ophthalmoscope) and were unable to demonstrate significant correlation between optic disc size and average or quadrant peripapillary RNFL thickness. It was hypothesized that large inter-individual variability in RNFL thickness and disc area within the population probably minimizes the effect of various ONH size on RNFL thickness measurement. Huang et al.^14^ found a similar result; in their study including 196 normal eyes, there was no significant association between RNFL thickness and optic disc area. In another study by Mansoori et al.,15 RNFL thickness and optic disc measurements were performed using spectral OCT/SLO in 102 normal subjects in the upper, average, and lower ranges of ONH size. In eyes with disc area <4 mm^2^, disc area did not affect RNFL thickness measurement. Average, superior and temporal quadrant RNFL thickness measurements were inversely proportional to disc area in eyes with disc area >4 mm^2^. The authors explained this by stating that RNFL fibers emerging from a large ONH must be distributed over a wider circumference and, as a consequence, the larger spatial distribution will result in a thinner RNFL when large ONHs are analyzed. In our study, the large ONH group comprised patients with ONH larger than 1.97 mm^2^. Mansoori et al.’s^15^ study included much larger ONHs compared to our study. We found a correlation between ONH size and RNFL thickness in the large ONH group in our study. However, contrary to our results, Mansoori et al.^15^ found far thinner RNFL in patients with an ONH larger than 4 mm^2^.

It is likely that the positive correlation between ONH size and RNFL thickness depends on the distance between the OCT circular scan and the ONH margin. If a fixed-diameter circular scan is employed, the distance between the scan and the ONH margin will be reduced in the presence of a large ONH. We are proposing this as an another theory to explain the varying nerve fiber thicknesses according to the ONH size at the fixed diameter of 3.4 mm via a resemblance to bicycle wheel spokes. The thickness of the RNFL measured at a fixed diameter of 3.4 mm may depend on the distances between the fibers on the circumference. In an eye with a small disc these distances between the fibers may be larger than those in an eye with a larger disc at that fixed circumference so that the thickness of RNFL in an eye with a large disc is measured thicker than the thickness of RNFL in an eye with a small disc.

## CONCLUSION

In conclusion, we showed that RNFL thickness as measured by Cirrus HD Spectral OCT is positively correlated with ONH size, and the differences in RNFL thickness between ONH size groups were statistically significant. This correlation and difference may arise due to a varying distance between the circular scan and the ONH margin. We believe that there is a need for studies that measure the RNFL thickness at a specific distance from the margin of the ONH regardless of varying ONH size.

## Ethics

Ethics Committee Approval: Başkent University Faculty of Medicine, Informed Consent: Obtained.

Peer-review: Externally peer-reviewed.

## Figures and Tables

**Table 1:**
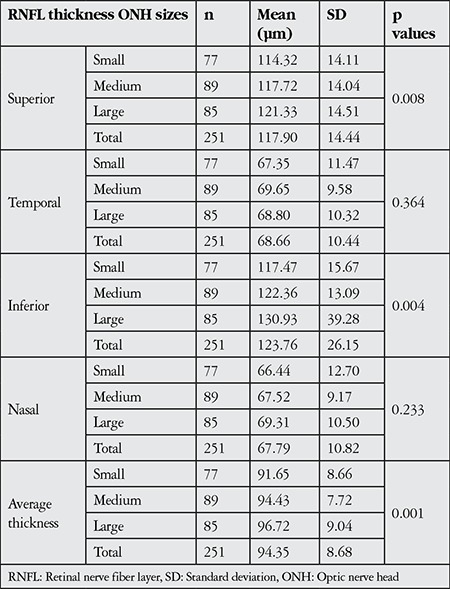
Comparison of mean retinal nerve fiber layer thickness values in optic disc size groups

**Table 2:**
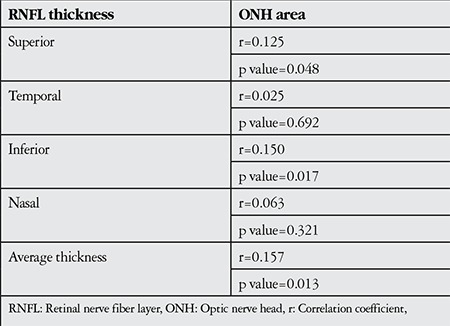
Correlation between retinal nerve fiber layer thickness and optic nerve head area
